# Ultraprocessed food, physical activity, and executive function: correlation and comparative study of university students in Mexico City and Salamanca

**DOI:** 10.3389/fpsyg.2025.1635050

**Published:** 2025-09-11

**Authors:** María Elena Chávez-Hernández, Lizbeth De La Torre, Luis Miguel Rodríguez-Serrano, Marina Wöbbeking-Sánchez

**Affiliations:** ^1^Facultad de Psicología, Universidad Anáhuac México, Huixquilucan, Estado de México, Mexico; ^2^Facultad de Psicología, Universidad Pontificia de Salamanca, Salamanca, Spain; ^3^Facultad de Psicología, Universidad de Salamanca, Salamanca, Spain

**Keywords:** ultraprocessed food, executive function, physical activity, university students, sitting time

## Abstract

**Introduction:**

Correlation of ultraprocessed food (UPF) intake on executive function (EF) and physical activity (PA) in university students has become a relevant subject of interest that remains insufficiently understood. PA has a positive impact on cognition and emotional status, while UPF intake has been associated with sedentary behavior and lower cognitive performance. The present study aims to evaluate the correlation between UPF intake, PA, and EF in university students by comparing the self-reports of Mexican and Spanish youth, as well as determine significant differences between both samples in the variables of interest.

**Methods:**

Undergraduate university students (*n* = 265, 18–25 years) who lived in Mexico City, Mexico and Salamanca, Spain, were included in the sample; a self-report online questionnaire was constructed including frequency of UPF intake, the International Physical Activity Questionnaire (IPAQ) Short Version and the Spanish version of the WEBEXEC Questionnaire.

**Results:**

Students in Mexico City show a significant positive correlation between PA and UPF intake, while Salamanca results indicate a significant correlation between sitting time and UPF intake, indicating that increased sedentary behavior (e.g. more sitting time or less PA) is related to increased UPF intake. Significant differences between both cities were found in sitting time and in UPF intake, while no statistically significant differences were found in EF and PA.

**Conclusion:**

The results of the present study provide initial indications of the relationship between UPF intake, physical activity, and sitting time, and executive functions in university students. This is a population in which the effect of these variables has not yet been thoroughly studied, even though the university lifestyle presents risk factors for unhealthy habits.

## 1 Introduction

Executive Functions (EF) are defined as a set of higher-order cognitive abilities that are necessary to examine and archive a goal (Cristofori et al., 2019), enable goal-directed behavior (Yang et al., 2018), support cognitive control (La Marra et al., 2022b) and allow inhibition of strong dominant responses/interfering stimuli (La Marra et al., 2022a). EF are essential cognitive skills that help regulate behavior and make it effective and socially acceptable (Ferguson et al., 2021; Ye et al., 2024). Consensus indicates that the three core EF are: (1) inhibition or inhibitory control, referring to the ability to suppress impulsive behavior, (2) cognitive flexibility, which is the ability to reconfigure the mind and switch between tasks, as well as develop different solutions for a particular problem, and (3) working memory, the ability to manipulate information temporarily (Diamond, 2013; Yang et al., 2018; Cortés Pascual et al., 2019; Cristofori et al., 2019). They are a set of mental skills responsible for controlling and regulating the behaviors, emotions, and cognitions needed to achieve goals, solve problems, and perform unlearned or non-routine actions (Albertos et al., 2021). Three EF developmental phases that extend from late childhood to adolescence have been identified, where brain plasticity is intensified in the development of executive control and are supported by hierarchically organized maturational changes in functional brain systems (Keller et al., 2023).

Furthermore, EF development has been shown to continue throughout adolescence and into early adulthood, and, in a university setting, will have a significant impact on student success during professional training (Ramos-Galarza et al., 2023) indicating that university is an equally important phase, where EF consolidate. In this sense, some evidence indicates an association between executive functioning and increased weight, with different patterns ascribed to individual differences (sex, age, lifestyles), suggesting that decision-making responses may differ in the overweight relative to the healthy weight condition (Favieri et al., 2022). Additionally, there are external factors that can influence this development, such as lifestyle, sleep habits, physical activity (PA), and/or eating habits (Forte et al., 2024) which is why it becomes relevant to explore how UPF intake and PA may relate to EF. Furthermore, some studies have found that there is a need to promote healthier lifestyle trends among medical students with a focus on diet and physical activity, and demonstrated improved EF; therefore, positive correlations between diet and physical activity with high academic achievement (Neuman et al., 2024).

Moderate-to-vigorous intensity exercise programs have been shown to improve cognitive performance and emotional status in general (Contreras-Osorio et al., 2022). Studies have shown that PA can improve performance on the EF tasks (Zhang et al., 2023) and that overall PA has a beneficial effect on EF in sedentary individuals, although the influence may be domain-specific and influenced by exercise prescription and age (Tian et al., 2023). Specific interventions for different life stages, such as university, should therefore be considered.

Additionally, a risk factor that has become relevant in recent years in young adult university students is overconsumption of ultra-processed foods (UPF). In this regard, reports indicate that young adult consume 68% of their total energy intake from UPF (Wang et al., 2021; Rego et al., 2023). Furthermore, the percentage of daily energy from UPF has been associated with cognitive decline in participants (Gomes Gonçalves et al., 2023). Also, increased neurobiological activation in areas of the brain implicated in EF (e.g., attention, planning, decision-making, inhibition), pleasure and reward experience, and sensory input and motor functioning has been associated with food according to its composition, and with UPF (Gordon et al., 2020). Additionally, research indicates that a higher percentage of daily energy consumption of UPF in adolescents (Dos Santos et al., 2024) and adults (Gomes Gonçalves et al., 2023) were associated with cognitive decline. Nevertheless, the relationship between UPF intake and cognitive performance remains unexplored in university students.

Lifestyles PA and access to quality food may also be influenced by the socioeconomic context. For example, there have been studies in the Chinese population examining the relationships between PA, body dissatisfaction, eating behavior, and depression in university students, which suggest that reducing body dissatisfaction and emotional eating holds significant potential for preventing depression and promoting general wellbeing (Yi et al., 2025). In the same vein, previous studies have used a food frequency questionnaire to assess dietary behavior, and the resulting data have been used to analyze the association between sugar-sweetened beverage and processed food consumption, physical health and depressive symptoms, finding that the more sugar-sweetened beverages and processed foods consumed, the worse the participants’ wellbeing (Xu et al., 2023).

Furthermore, Maltos-Gómez et al. (2024) report that university students from Mexico City show higher levels of depression associated with a frequent UPF intake, where daily UPF consumers have significantly higher levels of depression than weekly and occasional UPF consumers. Regarding PA and its association with UPF intake, in schoolchildren higher consumption of UPF has been associated with sedentary behavior (Oliveira et al., 2024). However, this relation has not yet been studied with university students.

Correlation of UPF consumption on EF and PA in university students has become a relevant subject of interest that remains insufficiently understood and presents as relevant given the rising global UPF intake and its potential consequences on cognition and overall health. Additionally, PA has been shown to have a positive impact on cognition and emotional status (Contreras-Osorio et al., 2022), and UPF intake has been associated with sedentary behavior in schoolchildren (Oliveira et al., 2024) and lower cognitive performance in adolescents (Dos Santos et al., 2024), while these associations remain unexplored in university students. Therefore, the present study aims to analyze the correlation between UPF intake, PA, and its impact on EF in university students by comparing the self-reports of Mexican and Spanish youth; also, we aim to evaluate significant differences between both samples in UPF intake, PA, and EF. Comparing these two cities can help comprehend how the correlation between these variables may differ in this population with different urban stressors, cultural norms and food environments.

## 2 Methods

### 2.1 Participants

The questionnaire was distributed to a sample of undergraduate college students, and snowball sampling was used as a non-probabilistic sampling technique. Our inclusion criteria were university students at the undergraduate level, aged 18–25 years old who lived in Mexico City, México, and in Salamanca, Spain, who agreed to participate voluntarily. The exclusion criteria for our study were participants outside of the age range (18–25 years old) and students who were not undergraduate level (graduate, diploma, etc.). People who did not wish to participate after reading the informed consent and invalid responses were also excluded; furthermore, non-valid (e.g., incomplete) questionnaire responses were eliminated and excluded from the analysis.

### 2.2 Materials and procedures

A cross-sectional correlational and comparative study was carried out in a group of undergraduate students from public and private universities within Mexico City, México, and Salamanca, Spain. In February 2025, undergraduate students were invited to participate in the study; informed consent was provided in accordance with guidelines from the National Bioethics Commission (Comisión Nacional de Bioética) of the Health Department of México (Secretaría de Salud de México) (Comisión Nacional de Bioética, 2014) and the 41/2002 Law that regulates the informed consent participation in Spain.

The students voluntarily agreed or disagreed to participate in the present study after being informed of the aim of the study, data analysis, and safekeeping, estimated time of participation, and ensuring that their participation would be anonymous. The final version of the questionnaire used to collect data included the following five sections: (1) informed consent; (2) sociodemographic data, including information regarding socioeconomic status (e.g., home characteristics, number of cars owned, internet access, etc.) to control this variable in the sample included.; (3) WEBEXEC Questionnaire; (4) International Physical Activity Questionnaire (IPAQ) Short Version; and (5) Ultraprocessed Food Frequency Questionnaire.

*WEBEXEC Questionnaire*. The Spanish version of the WEBEXEC Questionnaire was used to evaluate EF. It consists of six questions designed to assess global perception of executive function problems presented in a four-point Likert scale (0: I have no problems [No tengo problemas], 1: I have few difficulties [Tengo pocos problemas], 2: I have several difficulties [Tengo varios problemas], 3: I have many difficulties [Tengo muchos problemas]). It is a valid instrument with an overall Cronbach’s alpha of 0.85. Higher scores in the WEBEXEC questionnaire indicate more prevalence of problems with EF (Morea Domínguez and Calvete Zumalde, 2020). The WEBEXEC Questionnaire has been used to assess EF in multiple studies, indicating it external validity and prior use in research with similar populations (García-García et al., 2021; Bausela Herreras, 2022; Alvarenga et al., 2025).

*International Physical Activity Questionnaire (IPAQ) Short Version*. Physical activity was evaluated with the *International Physical Activity Questionnaire (IPAQ) Short Version*. It consists of seven items that measure self-reported frequency, duration, and moderate and high intensity PA in the last 7 days, as well as walking and sitting time during a workday. The IPAQ presents Intraclass Correlation Coefficient between 0.560 and 0.886, which indicates moderate to high reliability values. Two subscales were analyzed: IPAQ physical activity (IPAQ PA) and IPAQ sitting time (IPAQ ST) (Balboa-Castillo et al., 2023).

*Ultraprocessed Food Frequency Questionnaire*. To evaluate UPF intake, a frequency questionnaire including a list of foods classified as ultraprocessed according to the NOVA classification was used. Participants indicate intake frequency of each listed food during the last month with six answer options (0: never [nunca]; 1: 1 per month [1 vez al mes]; 2: 1–3 times per month [1 a 3 veces por mes]; 3: 1–3 times per week [1 a 3 veces por semana]; 4: 4–6 times per week [4 a 6 veces por semana]; 5: every day [todos los días]). Furthermore, a section regarding reasons to select these types of foods is included (Rodriguez, 2022).

### 2.3 Statistical analysis

Data obtained was prepared in Excel and analyzed using the statistical program Prism 9 for macOS (version 9.3.1; GraphPad Software LLC, San Diego, CA, USA). Normality tests (Kolmogorov–Smirnov) were performed, and correlation analysis (Spearman’s correlation, *r_s_*) was used to quantify the relationship between EF, IPAQ PA, IPAQ ST and UPF intake. Furthermore, Mann–Whitney’s U analysis was performed to compare the results from both samples in variables of interest. Significance level (*p*) was stablished in *p* ≤ 0.05.

## 3 Results

**Figure 1:**
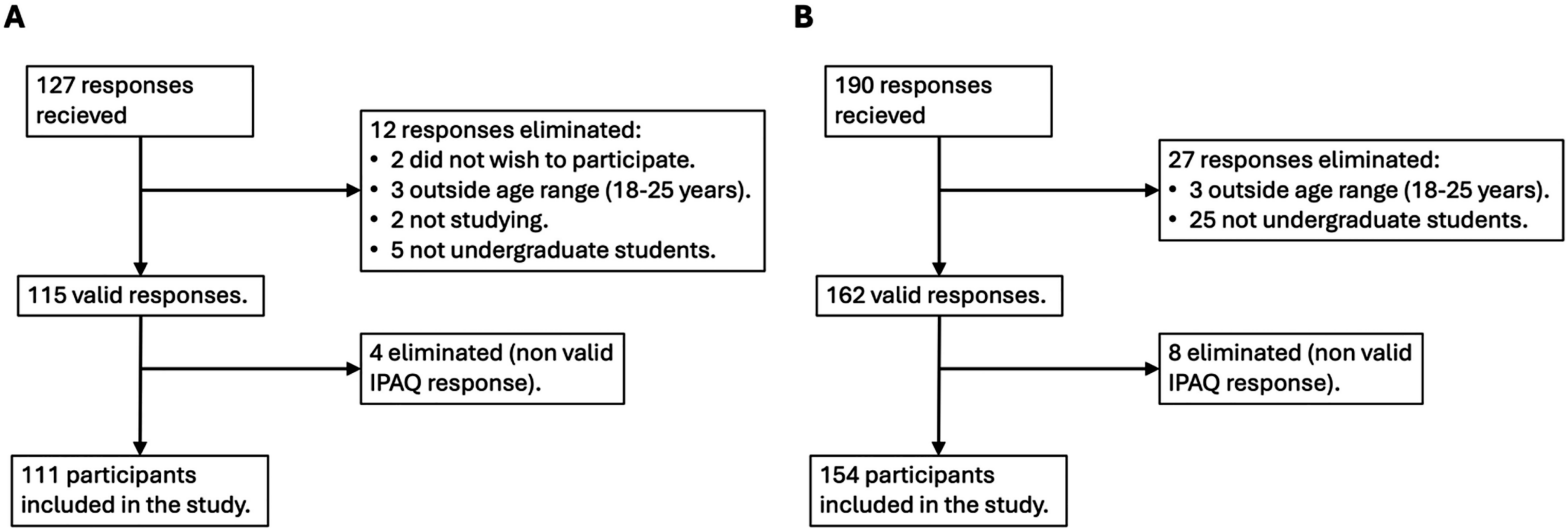
Flowchart of participants included in the final sample from Mexico City, Mexico **(A)** and Salamanca, Spain **(B)**. IPAQ, International Physical Activity Questionnaire.

A total of 317 responses were obtained (127 from Mexico City, Mexico and 190 from Salamanca, Spain). People who did not wish to participate after reading the informed consent (*n* = 2), participants who were outside of the age range (18–25 years old, *n* = 6), who did not study (*n* = 2) and who were not in undergraduate level (*n* = 30) were excluded; additionally, 12 participants were eliminated for non-valid responses in the IPAQ (see Figure 1). The final sample for the study consisted of 265 participants (111 for Mexico City, Mexico, and 154 for Salamanca, Spain), with a mean age of 19.47 (SD ± 1.69) years (20.00 mean age ± 1.80 for Mexico City, and 19.00 ± 1.50 for Salamanca). Table 1 shows the sample’s characteristics.

**Table 1:** Sample characteristics.

	Mexico City, Mexico	Salamanca, Spain
Total included, *n*	111	154
Women, *n* (%)	85 (76.58%)	119 (78.29%)
Men, *n* (%)	26 (23.42%)	35 (23.03%)
Mean age (SD)	20.00 (1.80)	19.00 (1.50)

Executive function	Mexico City, Mexico	Salamanca, Spain	Mann–Whitney’s U	*p*
WEBEXEC score, median	12.00	12.00	7,205	0.904
IPAQ, median				
IPAQ PA, average weekly mets	2,845.50	3,261.00	7,639	0.140
**IPAQ ST, average weekly sitting time (h)**	**2.50**	**3.50**	**6,786**	**0.0053**
Ultraprocessed foods intake, median				
Snacks and fast foods	1.31	1.29	8,281	0.756
Baked goods and refined cereals	1.33	1.33	8,375	0.779
Breakfast cereals and cookies	1.00	1.00	8,178	0.547
Candy	1.25	1.25	8,159	0.526
**Soups, salad dressings**	**1.17**	**1.50**	**6,327**	**0.0003**
Sugared beverages	1.33	1.67	7,489	0.0995
**Alcoholic drinks**	**1.00**	**2.00**	**4,573**	**<0.0001**

Bold values indicate significant differences between both samples. IPAQ PA, International Physical Activity Questionnaire, Physical Activity; IPAQ ST, International Physical Activity Questionnaire, Sitting Time.

Kolmogorov–Smirnoff tests were performed to assess normality of the sample, and results indicated that data did not pass normality tests (*p* < 0.0001). Therefore, for correlation analysis, Spearman’s rho was used to identify the relationship between EF, IPAQ PA, IPAQ ST, and UPF intake, and Mann–Whitney’s U analysis was performed to compare results between México City and Salamanca in variables of interest.

### 3.1 Correlation analysis

Table 2 presents Spearman’s correlation analysis results between the variables of interest in the Mexico City, Mexico and Salamanca, Spain samples.

**Table 2:** Spearman’s correlation analysis results.

	WEBEXEC	IPAQ PA	IPAQ ST
Mexico City’s correlation analysis results			
WEBEXEC	1.00	–	–
IPAQ PA	−0.01	1.00	–
IPAQ ST	0.06	0.10	1.00
UPF Snacks and fast foods	−0.12	−0.15	−0.01
UPF Baked goods and refined cereals	−0.04	−0.06	0.07
UPF Breakfast cereal and cookies	−0.06	−0.07	−0.02
UPF Candy	−0.09	−0.19*	0.09
UPF Soups, salad dressings	0.07	−0.12	−0.02
UPF Sugared beverages	0.00	−0.22*	0.15
UPF Alcoholic drinks	0.13	0.10	−0.02
Salamanca, Spain correlation analysis results			
WEBEXEC	1.00	–	–
IPAQ PA	−0.03	1.00	–
IPAQ ST	0.09	0.17*	1.00
UPF Snacks and fast foods	0.07	−0.06	0.08
UPF Baked goods and refined cereals	0.02	−0.08	0.09
UPF Breakfast cereal and cookies	−0.05	−0.04	−0.17*
UPF Candy	−0.02	0.01	0.04
UPF Soups, salad dressings	−0.03	−0.11	0.02
UPF Sugared beverages	0.10	0.05	0.17*
UPF Alcoholic drinks	0.12	−0.03	−0.02

Bold values indicate significant correlation between variables. IPAQ PA, International Physical Activity Questionnaire, Physical Activity; IPAQ ST, International Physical Activity Questionnaire, Sitting Time; UPF, Ultraprocessed Foods. Spearman’s correlation analysis. Asterisks indicate a significant correlation between variables *p < 0.05.

In Mexico City, significant negative correlations, with small effect sizes (measured by Spearman’s R correlation coefficient), were found with IPAQ PA and sugared drinks intake (*r_s_* = −0.22, *p* = 0.018) and with candy intake (*r_s_* = −0.19, *p* = 0.051), meaning that Mexico City students who report higher PA consume less sugared drinks and candy. No other significant correlations were found. Regarding correlation analysis from Salamanca, Spain, results indicate a small effect size significant negative correlation between IPAQ ST and breakfast cereal and cookies intake (*r_s_* = −0.169; *p* = 0.037), where students who report more weekly ST consume less breakfast cereals and cookies, and a small effect size significant positive correlation between IPAQ ST and sugared drinks intake (*r_s_* = 0.169; *p* = 0.038), where higher intake of sugared drinks is associated with more weekly sitting time.

### 3.2 Comparison between Mexico City and Salamanca

**Figure 2:**
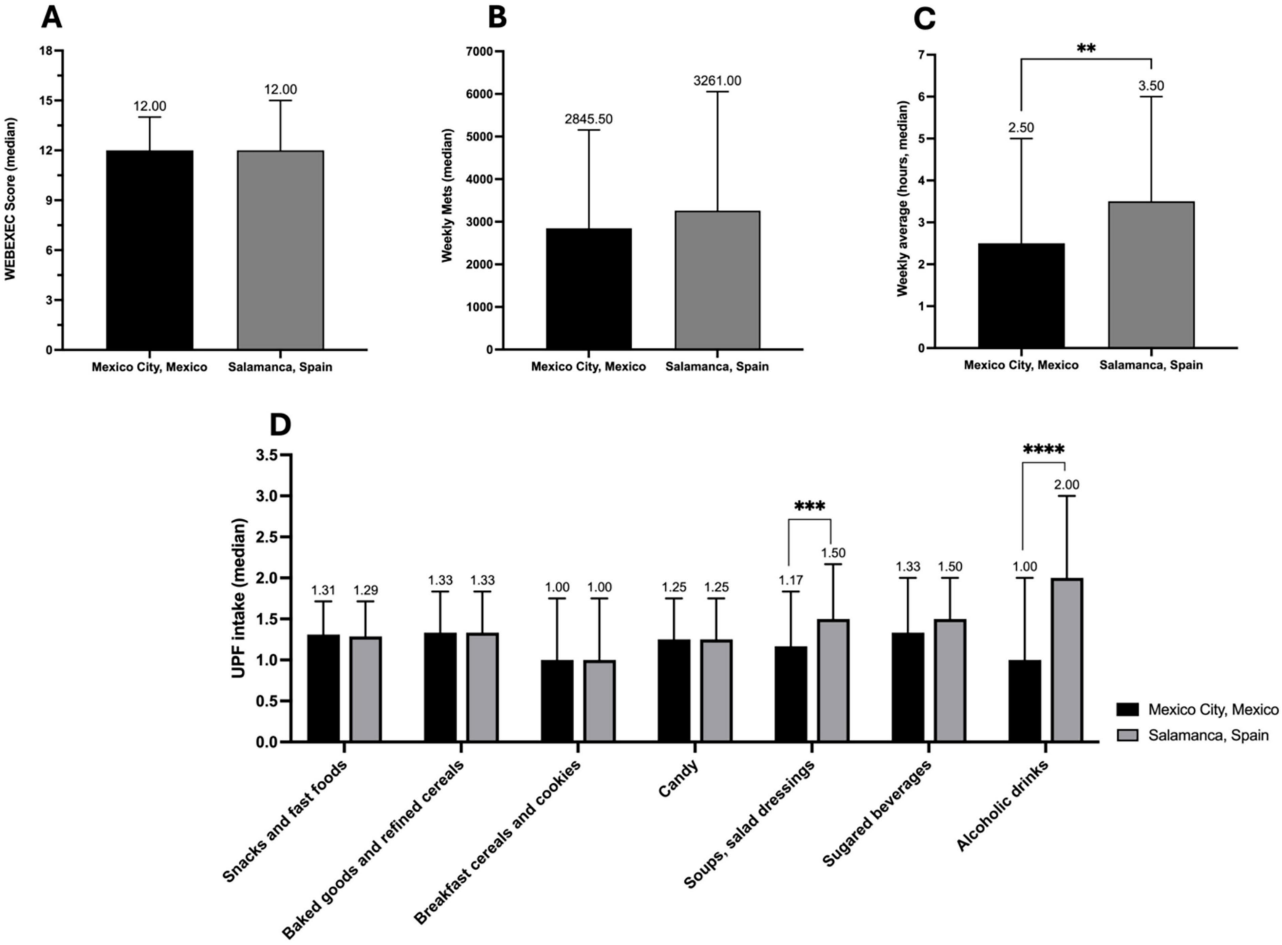
Comparison analysis between Mexico City and Salamanca, Spain. **(A)** WEBEXEC score results between the two samples. **(B)** IPAQ physical activity weekly mets results. **(C)** IPAQ weekly average sitting time (hours). **(D)** Average monthly and weekly ultraprocessed foods consumption results. Figures represent median values. Asterisks indicate a significant difference between samples; Mann–Whitney’s U analysis. **p < 0.01; ***p < 0.001; ****p < 0.0001.

To compare the results of EF, IPAQ PA, IPAQ ST, and UPF intake between Mexico City and Salamanca, a Mann–Whitney’s U analysis was performed. Results indicate a significant difference between the two samples in IPAQ ST (*U* = 6,786; *p* = 0.0053), where students from Salamanca spend on average more sitting time during a week (mean = 5.30 ± 7.60) than students from Mexico City (mean = 4.20 ± 5.60). Regarding UPF intake, a significant difference between both samples was found in soups and salad dressings (*U* = 6,327; *p* = 0.0003) with more intake from students in Salamanca (mean = 1.60 ± 0.79) than from Mexico City (mean = 1.20 ± 0.75), and in alcoholic drinks intake (*U* = 4,573; *p* < 0.0001), where students from Mexico City consume less alcoholic drinks (mean = 1.1 ± 0.99) than students from Salamanca (mean = 2.00 ± 1.00). No other significant differences were found between the samples (results are shown in Table 1 and in Figures 2A–D).

## 4 Discussion

The aim of the present study was to evaluate the correlation between consumption of UPF, PA, and EF in undergraduate university students from Mexico City and Salamanca. Statistical analysis revealed that there are significant correlations in both cities. Students from Mexico City showed a significant correlation between PA and UPF intake (sugared beverages and candy intake); meanwhile, the results from the Salamanca students indicated a significant correlation between ST and UPF intake (breakfast cereals and cookies, and sugared beverages). These results indicate that PA correlates with UPF intake in both samples, where increased PA reduces intake in students from Mexico City, while more ST correlates with UPF intake in students from Salamanca. Furthermore, when comparing results from both cities, significant differences were found in ST and in UPF intake (soups, salad dressings, and alcoholic drinks), whereas no differences were found regarding EF and PA. These findings indicate that university students from Mexico City and Salamanca differ in lifestyle indicators, while EF and PA remain stable in both samples.

Positive results have been observed regarding the performance of PA and its positive effect on EF, namely inhibitory control, planning, and cognitive flexibility. A relationship between variants of maximal oxygen consumption (VO_2_ max) and the practice of PA, along with performance on tests to assess cognitive function, suggesting a close relationship between high levels of PA and good performance in brain function (Maureira Cid, 2016). It should be noted that a sedentary lifestyle is a major risk factor for death, disease and/or disability (Han et al., 2022). In addition, it is an important factor in the premature development of cardiovascular risk in both childhood and adolescence, which is why attending to it at an early age is crucial and essential (Cantallops et al., 2012), which is why the child population should maintain a healthy relationship with PA (García Matamoros, 2019).

EF are believed to support child development across many domains of life, yet, future research should provide stronger causal tests of hypothesized relations between EF and outcomes to better understand the nature of EF (Stucke and Doebel, 2024). Thus, EF favor learning in all contexts, not only in an academic environment, and therefore it is essential to stimulate and address them as a whole to favor and promote the integral development of the individual (Donnelly et al., 2016). Finally, according to Moutoussamy et al. (2022) PA has beneficial effects on executive functions and episodic memory. Likewise, Maureira Cid (2016) states that with higher levels of PA, skills such as planning, cognitive flexibility, and/or inhibitory control are increased.

The sociocultural context of the population has been modified by the effects of globalization, which has promoted a change in lifestyles, including less time spent on buying and cooking food, as well as a transforming eating patterns and food preferences toward processed foods, leading to excessive consumption of foods of animal origin and refined sugars, thereby increasing saturated fats and cholesterol in the diet (Sánchez et al., 2017). According to the Pan American Health Association, increased sales of UPF have been associated with an increase in adult body mass index (BMI) (Pan American Health Organization, 2014). Observational studies have also demonstrated positive associations between UPF intake, weight gain, and overweight/obesity in adults (Dicken and Batterham, 2024). In this regard, several studies have shown that the type of diet is modified according to where students live, with university students, like the participants in the present study, who live in the family home having a greater adherence to the Mediterranean diet than those who live in student apartments (Navalón-Mira and Fabregat-Cabrera, 2022). In this sense, the university phase is crucial because it is a time of transition and change, influenced by the new ways in which young people live together and the new responsibilities they assume, including in food habits, as well as academic demands. Studies have shown that college students are faced with a very demanding lifestyle where they may not have enough time for physical activity, which is essential for overall health, and/or a balanced diet, increasing the consumption of UPF (Larcom et al., 2024). On the other hand, it has been found that the list of problematic foods increases among students with food addiction, mainly due to products with a high carbohydrate content, including UPF (Borisenkov et al., 2021). This may be an obstacle to achieving optimal overall health, in addition to having a long-term impact on executive functions, which are essential cognitive skills such as behavior regulation, decision making, among others (Gordon et al., 2020).

The present study analyzed the relationship between UPF consumption, PA, and EF in undergraduate college students aged 18–25 from Spain and Mexico. Previous studies with the Mexican population have shown that a high number of food addiction symptoms were associated with higher executive dysfunction scores, greater reward sensitivity, and more severe depressive and binge eating problems; therefore, more processed food intake (Téllez-Rodríguez et al., 2023). Furthermore, results from Salamanca students show a non-significant positive correlation where higher EF difficulties is associated with increased snacks and fast foods intake, while in students from Mexico City a non-significant negative correlation was found between EF difficulties with snacks and fast foods intake. In this regard, it has been shown that executive function is positively correlated with healthy food intake in university students from Mexico City (Chavez-Hernandez, 2023). These findings suggest that there is a trend toward an association between a healthy lifestyle (physical activity and a balanced diet) and a positive impact on EF. Both samples show a non-significant positive correlation between alcoholic drinks intake and EF difficulties.

Our results from Mexico City also indicate there is a negative correlation between PA and UPF intake, while results from Salamanca indicate a negative correlation between sitting time (sedentary behavior) and UPF intake in students, which has been previously reported in schoolchildren (Oliveira et al., 2024). Additionally, it remains to be seen what significant differences can be found in the different socio-demographic contexts of university students (Spain–Mexico). Regarding the Spanish population, no studies so far have looked specifically at this issue, but there are other studies that have found that eating habits are influenced by ethnicity (De Vito et al., 2025).

The UPF refers to foods with industrial formulations characterized by their extensive processing and use of industrial ingredients and cosmetic additives (Louie, 2025), also it is high average energy density and nutrient-poor composition (Medin et al., 2025). Furthermore, the UPF the fourth group in the NOVA classification system (Sun et al., 2024). The UPF group includes packaged snacks, sweetened beverages, ready-to-eat meals, and processed meat products (Fardet, 2024). In this regard the UPF questionnaire used in the present study uses a list of foods classified as UPF, avoiding bias in nutritional or cultural relevance in both samples.

The present study presents some limitations that should be considered. First, UPF intake was assessed with a self-reported frequency questionnaire, which may be vulnerable to recall bias and inaccuracies given that participants may report underconsumption or lack awareness of what constitutes an UPF. Second, cross-sectional study designs such as the present one limit the ability to detect potential long-term effects of UPF on EF, as these may present and/or accumulate in the long term. Despite this, results from the present study show some first indicators of the relation UPF intake has on PA and sitting time lifestyle in university students, a population in which the effect of these variables has not yet been thoroughly studied, even though university lifestyle presents risk factors to unhealthy habits. Future studies with longitudinal approaches may help further elucidate these correlations, as well as the relation with EF, by assessing in different timepoints UPF intake, PA and EF in university students throughout their career. Finally, snowball sampling might be considered a limitation given that this is a non-probabilistic sampling technique used when it is difficult to access a specific population for a study. Using random sampling in future studies could provide more information in the association of UPF, EF and PA. Overall, our results indicate that UPF intake is related to lifestyle PA factors in university students from Mexico City and Salamanca, and that students from both cities do not significantly differ in EF, but lifestyle elements such as sitting time and UPF intake are different in both samples. Given that university students present as a risk population in UPF intake and low PA, university policies should consider addressing this issue by promoting healthy foods in their cafeteria and sale establishments, as well as promote PA. In this regard, Mexico recently approved the prohibition of UPF sales within schools, a policy that could also be applied in university environments worldwide (UNICEF, 2024).

## Data Availability

The raw data supporting the conclusions of this article will be made available by the authors, without undue reservation.
