# Supplementary material for: Ultraprocessed food, physical activity, and executive function: correlation and comparative study of university students in Mexico City and Salamanca
Source: Front Psychol. 2025 Sep 11;16:1635050. doi: 10.3389/fpsyg.2025.1635050 (PMC12460331; doi:10.3389/fpsyg.2025.1635050)
Supplement: Supplementary file 1 [file Data_Sheet_1.PDF]

## Supplementary Material

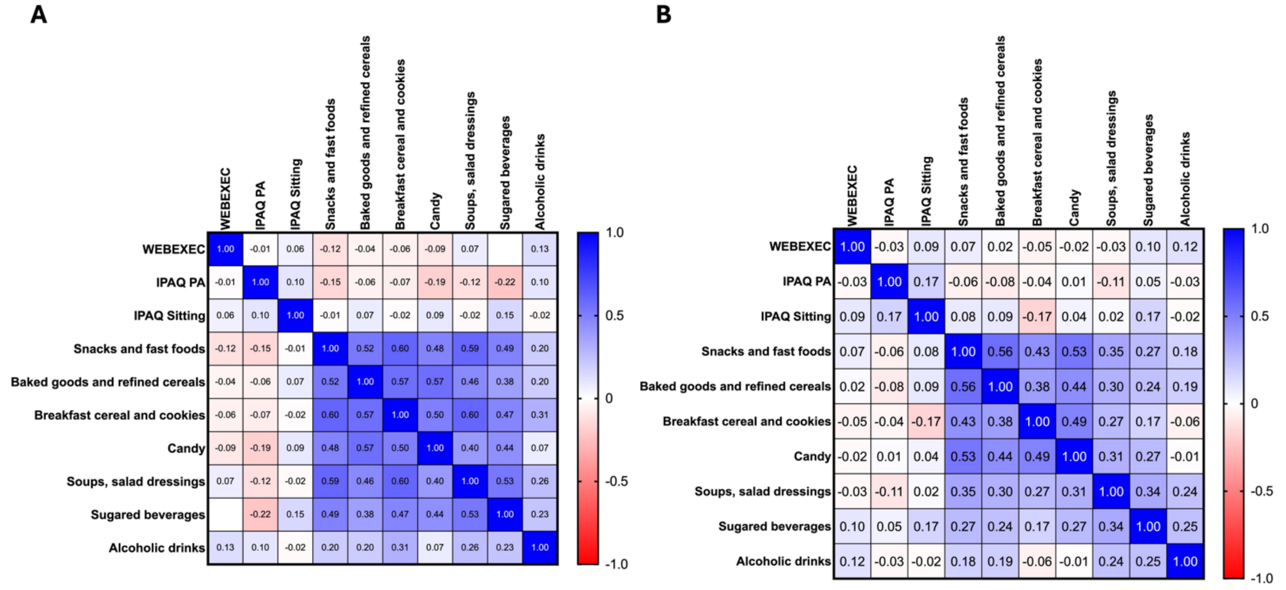


Supplementary Figure 1: Spearman's correlation analysis between variables of interest. A: Mexico City, Mexico, correlation analysis results. B: Salamanca, Spain correlation analysis results. IPAQ PA: International Physical Activity Questionnaire, Physical Activity. IPAQ Sitting: International Physical Activity Questionnaire, Sitting Time.
